# 五次应用分子靶向药物治疗晚期肺癌患者1例

**DOI:** 10.3779/j.issn.1009-3419.2013.06.11

**Published:** 2013-06-20

**Authors:** 万峰 郭, 红军 高, 晓晴 刘

**Affiliations:** 100071 北京，军事医学科学院附属医院肺部肿瘤内科 Department of Lung Cancer, Afliated Hospital, Academy of Military Medical Sciences, Beijing 100071, China

目前，表皮生长因子受体酪氨酸激酶抑制（epidermal growth factor receptor tyrosine kinase inhibitor, EGFR-TKI）在晚期非小细胞肺癌（non-small cell lung cancer, NSCLC）的治疗中取得了较好的疗效。但是，EGFR-TKI如何应用取得最佳治疗效果仍是亟待解决的问题。临床上针对EGFR-TKI明显获益的患者反复应用EGFR-TKI药物，可能取得较好的疗效。

## 1 临床资料

患者女性，58岁，无吸烟史。2007年6月无明显诱因出现咳嗽、咳痰，无喘憋及呼吸困难。7月2日于北京某医院行右锁骨上淋巴结穿刺，病理发现癌细胞，考虑腺癌细胞。明确诊断为右肺腺癌Ⅲb期。一线口服吉非替尼250 mg，每日1次，最佳疗效为部分缓解（partial remission, PR），无疾病进展时间（progression-free survival, PFS）为15个月。2008年10月24日二线行培美曲噻+顺铂化疗6周期，最佳疗效为稳定（stable disease, SD），PFS为6个月。病情进展后2009年4月入军事医学科学院附属医院进一步治疗。胸部CT提示右肺癌，右侧胸腔积液（图 1A）；行胸水EGFR基因突变检测，提示外显子19序列缺失；外显子21未见点突变（图 2A）。*K-ras* 12、13号密码子均未见突变。三线再次口服吉非替尼250 mg，每日1次，最佳疗效为SD，PFS为6个月。2009年10月9日胸部CT提示病情进展，于10月16日始行紫杉醇和顺铂四线化疗6周期，最佳疗效为PR，PFS为7个月。2010年5月入院复查提示病情进展，5月15日开始五线口服盐酸厄洛替尼150 mg，每日1次。2010年6月入院复查胸部CT提示病情进展，右侧胸腔积液，行胸腔积液穿刺引流术，共引流1, 700 mL淡黄色积液。再次行胸水*EGFR*基因突变检测，外显子19和21未见突变，外显子20未见T790M突变（图 2B）。遂停止口服厄洛替尼靶向治疗。2010年7月5日开始吉西他滨和卡铂方案六线化疗6周期，最佳疗效为SD，PFS为6个月。2011年1月04日和1月25日行七线单药培美曲噻方案化疗2周期，疗效评价为病情进展（progression disease, PD）。患者肿瘤进展符合BIBW2992药物临床研究。4月21日进行随机分组，开始口服研究药物。6周疗效评价为PR，12周疗效评价为PD。患者于7月22日和8月18日九线行多西他赛方案单药化疗2周期。2011年9月复查胸部CT，患者病情进展。头颅MRI示小脑蚓部转移伴少量出血。9月20日行小脑蚓部转移灶伽玛刀治疗。患者10月11日十线再次口服吉非替尼375 mg（1次/d）治疗，PFS为4月余，最佳疗效评价为SD。患者2012年2月20日再次返院，病情进展（图 1B）。患者于2012年4月死亡。

**1:**
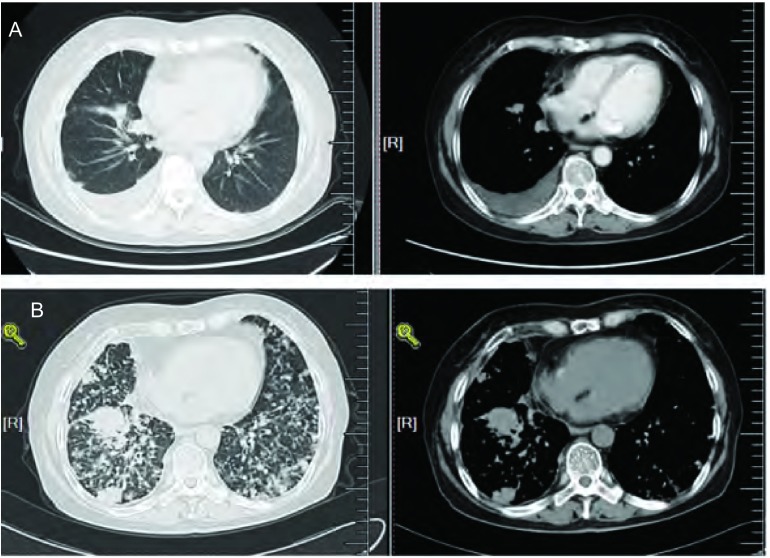
患者治疗过程中的CT结果。A：2009年4月患者入我院时的胸部CT示右肺癌和胸腔积液；B：2012年2月患者胸部CT示右肺癌伴广泛转移。 CT scan of the chest during the therapy. A: CT scan of the chest showing a soft tissue mass and pleural effussion at right lung; B: CT scan of the chest showing right lung caner and extensive metastasis.

**2:**
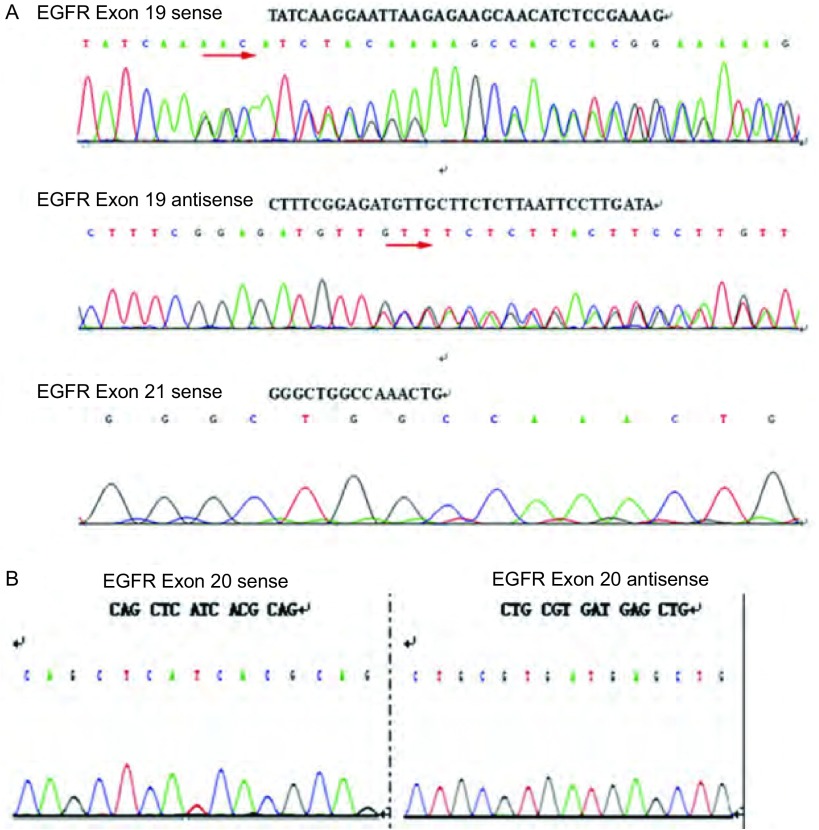
患者*EGFR*基因测序结果。A：*EGFR*基因外显子19序列缺失；外显子21未见点突变；B：*EGFR*外显子20未见T790M突变。 *EGFR* gene sequencing of the patient. A: Sequence deletion is detected in the EGFR exon 19 and no gene mutation is detected in the EGFR exon 21; B: No gene mutation is detected in the EGFR exon 20. EGFR: epidermal growth factor receptor.

## 2 讨论

EGFR-TKI已经成为晚期NSCLC不可或缺的治疗方法，取得了非常好的疗效^[1]^。NCCN指南推荐其用于晚期NSCLC的二线、三线治疗以及*EGFR*基因敏感型突变患者的一线治疗^[2, 3]^。EGFR-TKI治疗失败后的后续治疗选择是目前临床关注的热点。关于EGFR-TKI治疗失败后重复使用EGFR-TKI患者再次获益的研究多为回顾性、小样本研究^[4-7]^。Kaira等^[4]^对11组研究共106例吉非替尼失败后厄洛替尼治疗的患者进行了汇总分析，二次EGFR-TKI治疗的PR、SD、疾病控制率（disease control rate, DCR）分别为9.9%、18.9%和28.8%。国内文献^[5]^报道首次TKI治疗的71例晚期NSCLC患者失败后再次使用EGFR-TKI，PR、SD、DCR分别为7%、36.6%和56.3%。然而，对于EGFR-TKI该数据显示接受EGFR-TKI重复治疗至少有30%-50%的患者临床受益。单因素分析显示首次EGFR-TKI缓解期≥6个月、两次EGFR-TKI间隔期≥3个月的患者从再次应用EGFR-TKI中获益的机率更大。而Murakami等^[6]^对于90例使用厄洛替尼的患者进行分析，研究认为22例应用过吉非替尼的患者比没有应用过吉非替尼的患者的中位生存时间缩短（283天*vs* 177天），但是差异没有统计学意义（*P*=0.329）。单变量分析显示应用吉非替尼疾病进展时间1年以上是一个独立预后指标。Hata等^[7]^研究了2008年1月-2009年5月的125例吉非替尼治疗失败后应用厄洛替尼的患者，分析认为PS状态、*EGFR*突变阳性和从既往吉非替尼获益的状况是疾病控制的预测因素。该患者一般状况较好，*EGFR*基因外显子19序列缺失，一线应用吉非替尼，PFS达15个月，预示了该患者可能有较好的预后。因此，在4年10个月的病程中先后5次使用EGFR-TKI类药物治疗，累计获益时间为30个月。

BIBW2992（afatinib，阿法替尼）是第二代高效双重非可逆性的酪氨酸激酶抑制剂，同时抑制EGFR和HER-2两种受体，是首个用于EGFR抑制剂治疗失败后肺癌患者的药物。IIb/Ⅲ期试验结果显示，585例晚期NSCLC患者服用afatinib后无进展生存期比安慰剂组延长（3.3个月*vs* 1.1个月，*P* < 0.000, 1），而且提高客观缓解率及DCR^[8]^。该患者经过反复EGFR-TKI治疗和化疗后，八线口服研究药物BIBW2992，6周疗效评价为PR，12周为PD，PFS为3个月。

吉非替尼的临床使用剂量是250 mg/d，低于其最大耐受剂量（1, 000 mg/d）。研究^[9]^表明，常规用量下脑脊液内吉非替尼浓度远远低于血液内浓度，但对于脑转移患者血脑屏障破坏，脑脊液内药物浓度提高，临床可通过加大用量提高疗效。因此，对于吉非替尼耐药患者加大吉非替尼用量，有望再次使患者从中获益。此患者既往从吉非替尼治疗中获益时间达21个月，同时为了兼顾脑转移，提高脑脊液内药物浓度，十线给予吉非替尼加量治疗（375 mg 1次/d），患者再次从中获益4月余。

因此，对于EGFR-TKI靶向治疗明显获益的NSCLC患者，临床可以考虑多次应用EGFR-TKI靶向治疗，使生存获益。当然，这只是1例个案，需要更多的经验和临床实践。
